# FES avid pulmonary adenocarcinoma and confounding ER+ breast carcinoma

**DOI:** 10.1016/j.radcr.2024.10.114

**Published:** 2024-11-14

**Authors:** Yousif M. Abdelmoneim, Yanyan Lou, Pooja Advani, Andras Khoor, Akash Sharma, Ephraim E. Parent

**Affiliations:** a Department of Oncology, Mayo Clinic Florida, FL, 32224, USA; b Department of Pathology, Mayo Clinic Florida, FL, 32224, USA; c Department of Radiology, Mayo Clinic Florida, FL, 32224, USA

**Keywords:** Estrogen receptor, FES, Pulmonary adenocarcinoma, Breast carcinoma

## Abstract

Breast carcinomas are well known for expression of estrogen receptor (ER) however there are other malignancies that are also express ER, possibly confounding the diagnostic interpretation of 16α-[^18^F]fluoro-17β-estradiol (FES; Cerianna GE HealthCare) in patients with both ER+ breast carcinomas and other malignancies. We present a case of a woman with prior history of both ER+ breast carcinoma and pulmonary adenocarcinoma with subsequent identification of an FES pulmonary nodule that was proven on histopathology to be consistent with an ER expressing pulmonary adenocarcinoma metastasis

## Introduction

16α-[^18^F]fluoro-17β-estradiol (FES; Cerianna GE HealthCare) was approved by the FDA for clinical use in 2020, and remains the only approved nuclear receptor imaging agent for patients with estrogen receptor positive (ER+) breast carcinoma. FES PET is routinely utilized to guide management in patients with ER+ breast carcinoma by noninvasively providing whole body interrogation of bioavailable ER+ disease, prognosticating response to estrogen targeted therapies, and helping distinguish ER+ breast carcinoma metastases from other malignancies when present. Here we present a case of a woman with an FES avid pulmonary nodule that was incorrectly identified as a ER+ breast carcinoma metastasis but on histopathology was found to be an ER+ pulmonary adenocarcinoma.

## Case report

**Fig. 1:**
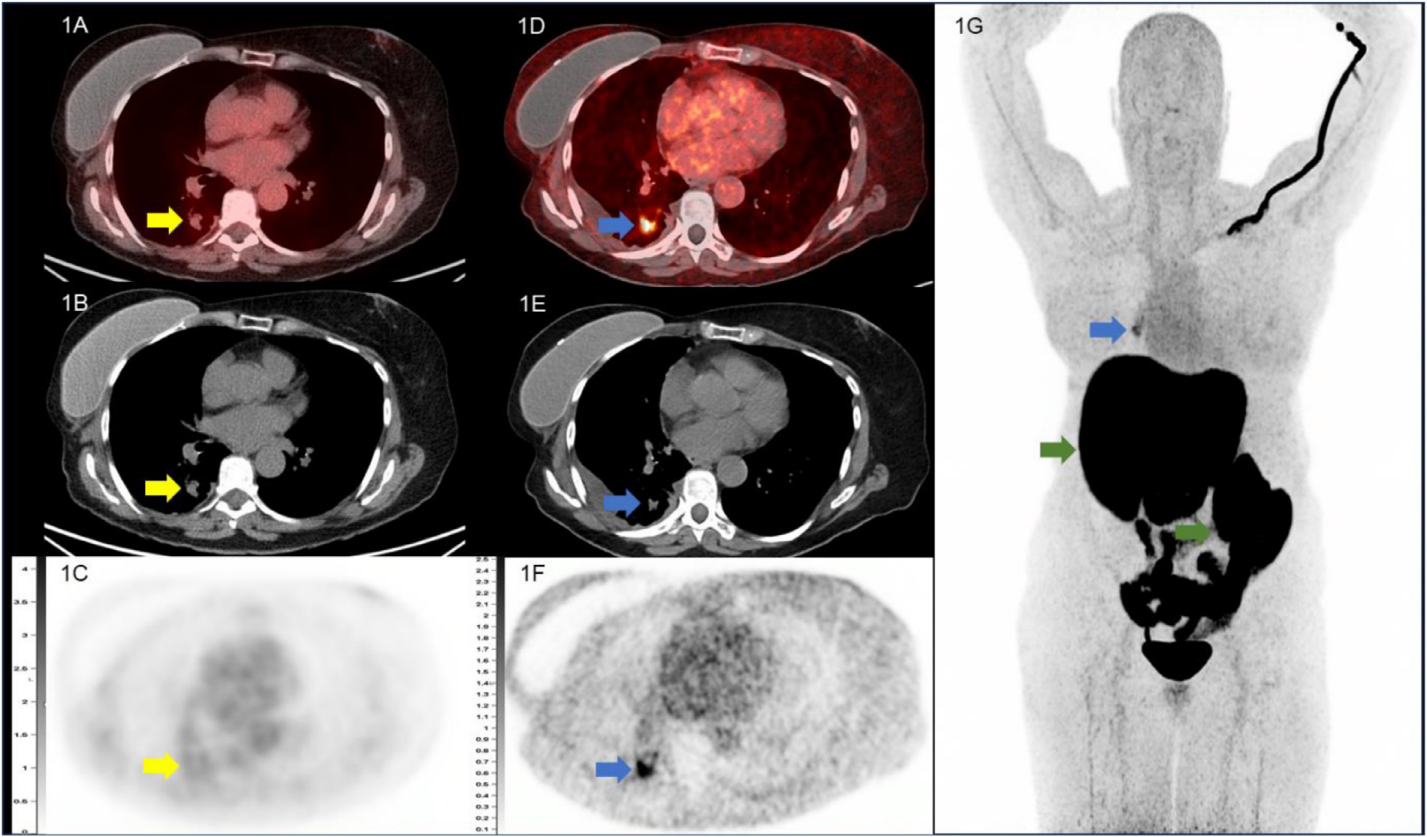
Selected transaxial images from the initial FDG PET/CT and subsequent FES PET/CT in this patient with enlarging pulmonary nodule and prior ER+ breast cancer and pulmonary adenocarcinoma. Transaxial fused FDG PET/CT (1A) CT (1B) and FDG PET (1C) images show a right lower lobe pulmonary nodule with uptake comparable to blood pool with SUVmax 2.5 (yellow arrow). Transaxial fused FES PET/CT (1D) CT (1E) and FES PET (1F) images demonstrate avid uptake in the pulmonary nodule with SUVmax 3.0 (blue arrow). Coronal FES PET maximum intensity projection (MIP; 1G) demonstrates the FES avid pulmonary nodule which was biopsy proved pulmonary adenocarcinoma (blue arrow) and no other abnormal FES avid foci to suggest additional ER+ disease, either from lung or breast primary. Note intense physiological FES uptake in the liver and excretion through the biliary tree into the bowel (green arrow).

**Fig. 2:**
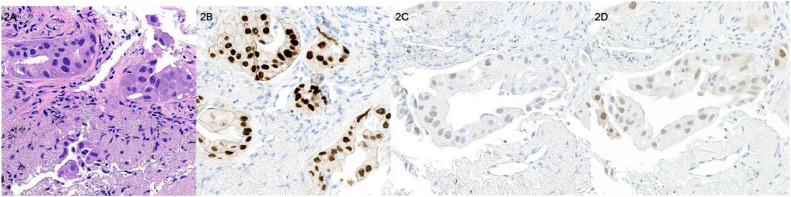
Histologic sections of the right lower lobe cryobiopsy demonstrate adenocarcinoma with acinar growth pattern (Fig. 2A) and with immunohistochemical analysis positive for TTF-1 (Fig. 2B) and negative for p40, GATA-3, and BRST-2, consistent with an adenocarcinoma of lung origin. In additional immunohistochemical studies, approximately 50% of the tumor cells exhibited weak positivity for estrogen receptor and progesterone receptor (Figs. 2C and 2D). Original magnification x630.

A 71-year-old woman with previously treated breast carcinoma and lung adenocarcinoma was found to have an enlarging pulmonary nodule suspicious for malignancy on standard of care follow up imaging. Her right breast invasive ductal carcinoma (ER+, PR+, HER-2 −) was diagnosed 10 years prior to presentation and had undergone definitive chemoradiation therapy at that time and subsequently maintained on aromatase inhibitor. Afterwards, she was also diagnosed with a right upper lobe pulmonary adenocarcinoma (EGFR+) 3 years prior to presentation and treated with lobectomy, radiation therapy, and maintained on Osimertinib (Tagrisso) until current presentation. Tumor biomarkers CA 27-29 and CA 15-3 were both found to be concurrently rising at time of pulmonary nodule identification (CA 27-29 of 44.15 U/mL and CA 15-3 of 50.3 U/mL) and no other sites of possible disease were identified on traditional imaging to help elucidate the primary etiology of the presumed metastatic deposit. A 2-deoxy-2-[^18^F]fluoro-D-glucose (FDG) positron emission tomography (PET) / computed tomography (CT) scan was obtained for aid in diagnosis and staging, and which demonstrated mildly increased FDG uptake in the pulmonary nodule comparable to blood pool (Fig. 1A) and no other potential sites of hypermetabolic disease. Subsequently, the patient underwent 16α-[^18^F]fluoro-17β-estradiol (FES) PET/CT in the expectation that FES uptake in the pulmonary nodule would be selective and confirmatory for any potential estrogen receptor positive (ER+) breast cancer metastatic deposits, and thus allowing for proper staging and treatment. FES PET/CT demonstrated high level of FES uptake in the pulmonary nodule and was interpreted as ER+ breast metastatic pulmonary disease (Fig. 1D). As no other sites of disease were identified, the patient underwent endobronchial ultrasound guided biopsy of the pulmonary nodule for definitive diagnosis with histopathology findings consistent with pulmonary adenocarcinoma recurrence (Figs. 2A and B). Additional immunohistochemical studies demonstrated approximately 50% of the tumor cells exhibited weak positivity for ER and progesterone receptor (PR) (Figs. 2C and D).

## Discussion

17-β-Estradiol (E2) is the primary reproductive hormone in females, and it is synthesized under the stimulation of FSH and LH. Other estrogens such as estriol and estrone are mainly metabolites of E2 and synthesized in the liver [1]. ER receptors compromise 2 types: ER-α and ER-β and each are products of different genes (ER-α resides on chromosome 6 and ER-β on chromosome 14). While ER-α expression is mainly in breast, ovarian and endometrial tissue, ER-β is widely distributed throughout the body including lung, kidney, brain, colon, bone, prostate, testes, ovaries, and endothelial cells [2].

When estrogen and its analogs (E2, estriol or estrone) bind to ER, they activate genomic and nongenomic pathways; genomic pathways induce transcription and nongenomic activation involves ER translocation to the cell membrane leading to rapid protein kinase activation and regulation of ion channels [1]. ER-β receptors are highly expressed in the pulmonary bronchial epithelial cells and pneumocytes and are important for the maintenance of the extracellular matrix, with ER-β null mice having lower numbers of alveoli, decreased surfactant, and platelet derived growth factor A. Additionally, other studies have shown ER-α affects alveolar number and surface area, and ER-β affects lung elastic tissue recoil [2]. Lung carcinomas have been shown to synthesize estrogens and Beattie et al reported that up to 30% of cells in pulmonary squamous and adenocarcinomas have express ER [3]. Another study similarly reported that approximately 18% of nonsmall cell lung carcinomas express ER [4]. Despite the relatively small number, these studies suggest that ER and estrogen activation may have a role in lung carcinogenesis.

*In vitro* nonsmall cell lung cancer studies have shown that E2 stimulates tumor cells’ division [5], and also enhances the expression of genes linked to tumor progression including: VEGF secretion, and activation of MAPK, Akt, and CREB through phosphorylation. Additionally, it was found that women on estrogen replacement therapy have higher risk of developing lung adenocarcinoma and early menopause ≤ 40 years may be protective with a decreased risk of developing lung adenocarcinoma in this population [5]. While the Women's Health Initiative Trial found that hormonal replacement therapy (HRT; combined estrogen and progestin) did not increase the incidence of lung cancer, it did increase the risk of dying from nonsmall cell carcinoma in postmenopausal women by 60% [6]. ER-β is the predominant form of ER expressed in lung cancer [7], and while nuclear ER-β expression is associated with better prognosis [8], ER-α expression alone or in existence of ER-β is associated with a worsened prognosis [1].

16α-[^18^F]fluoro-17β-estradiol (FES) was approved by the FDA for clinical use in 2020 for “PET imaging for the detection of ER+ lesions as an adjunct to biopsy in patients with recurrent or metastatic breast cancer” [9]. FES has a nanomolar range binding affinity for both ERα and ERβ, and while FES has a preferential affinity for ERα, it is unable to distinguish between ERα and ERβ on clinical studies [10]. ERβ specific radiopharmaceuticals has been developed but have not yet been evaluated in human studies [[10], [11], [12], [13]]. To the best of our knowledge, this is the first report of FES avid disease in ER+ pulmonary adenocarcinoma and may have clinical implications as a unique means to identify ER expressing pulmonary adenocarcinoma and to help guide potential new hormonal therapies.

## Conclusions

This case is noteworthy in not just the unique presentation of ER+ pulmonary adenocarcinoma as identified on FES PET/CT but also in the potential utilization of FES PET to prognosticate responders from nonresponders in future ER targeted clinical trials. Additionally, while FES PET can be used to help distinguish ER+ breast carcinoma metastasis from other malignancies, there are several other diseases that can be ER+ and the interpreting physician should familiarize themselves with the potential for mistaken identification.

## Declaration of generative AI and AI-assisted technologies in the writing process

No use of AI technology was used in the preparation of this manuscript.

## Patient consent

Written informed permission has been obtained for the publication of this case report

## References

[1] Hsu L.H., Chu N.M., Kao S.H. (2017). Estrogen, estrogen receptor and lung cancer. Int J Mol Sci.

[2] Baik C.S., Eaton K.D. (2012). Estrogen signaling in lung cancer: an opportunity for novel therapy. Cancers (Basel).

[3] Beattie C.W., Hansen N.W., Thomas P.A. (1985). Steroid receptors in human lung cancer. Cancer Res.

[4] Cagle P.T., Mody D.R., Schwartz M.R. (1990). Estrogen and progesterone receptors in bronchogenic carcinoma. Cancer Res.

[5] Bogush T.A. (2010). Estrogen receptors, antiestrogens, and non-small cell lung cancer. Biochemistry (Mosc).

[6] Chlebowski R.T. (2009). Oestrogen plus progestin and lung cancer in postmenopausal women (women's health initiative trial): a post-hoc analysis of a randomised controlled trial. Lancet.

[7] Zhang G. (2009). Estrogen receptor beta functions through nongenomic mechanisms in lung cancer cells. Mol Endocrinol.

[8] Wang Z. (2015). ERbeta localization influenced outcomes of EGFR-TKI treatment in NSCLC patients with EGFR mutations. Sci Rep.

[9] *United states food and drug administration.* Drug Trial Snapshot: CERIANNA. [accessed 10.10.2022]; https://www.fda.gov/drugs/drug-approvals-and-databases/drug-trial-snapshot-cerianna.

[10] Yoo J. (2005). Synthesis of an estrogen receptor beta-selective radioligand: 5-[18F]fluoro-(2R,3S)-2,3-bis(4-hydroxyphenyl)pentanenitrile and comparison of in vivo distribution with 16alpha-[18F]fluoro-17beta-estradiol. J Med Chem.

[11] Moon B.S. (2009). Synthesis and evaluation of aryl-substituted diarylpropionitriles, selective ligands for estrogen receptor beta, as positron-emission tomographic imaging agents. Bioorg Med Chem.

[12] Lee J.H. (2012). Synthesis and biological evaluation of two agents for imaging estrogen receptor β by positron emission tomography: challenges in PET imaging of a low abundance target. Nucl Med Biol.

[13] Antunes I.F. (2017). Synthesis and evaluation of the estrogen receptor β-selective radioligand 2-(18)F-fluoro-6-(6-hydroxynaphthalen-2-yl)pyridin-3-ol: comparison with 16α-(18)F-fluoro-17β-estradiol. J Nucl Med.

